# High-Speed and Hysteresis-Free Near-Infrared Optical Hydrogen Sensor Based on Ti/Pd Bilayer Thin Films

**DOI:** 10.3390/nano15141105

**Published:** 2025-07-16

**Authors:** Ashwin Thapa Magar, Tu Anh Ngo, Hoang Mai Luong, Thi Thu Trinh Phan, Minh Tuan Trinh, Yiping Zhao, Tho Duc Nguyen

**Affiliations:** 1 Department of Physics and Astronomy, University of Georgia, Athens, GA 30602, USA; anhngo@uga.edu (T.A.N.); zhaoy@uga.edu (Y.Z.); 2 Department of Electrical Engineering, Faculty of Engineering, Chulalongkorn University, Bangkok 10330, Thailand; hoang.l@chula.ac.th; 3 Department of Chemistry and Biochemistry, Utah State University, Logan, UT 84322, USA; phan.trinh@usu.edu (T.T.T.P.); t.trinh@usu.edu (M.T.T.)

**Keywords:** optical hydrogen sensor, near-infrared (NIR) sensing, titanium, palladium, bilayer, thin film

## Abstract

Palladium (Pd) and titanium (Ti) exhibit opposite dielectric responses upon hydrogenation, with stronger effects observed in the near-infrared (NIR) region. Leveraging this contrast, we investigated Ti/Pd bilayer thin films as a platform for NIR hydrogen sensing—particularly at telecommunication-relevant wavelengths, where such devices have remained largely unexplored. Ti/Pd bilayers coated with Teflon AF (TAF) and fabricated via sequential electron-beam and thermal evaporation were characterized using optical transmission measurements under repeated hydrogenation cycles. The Ti (5 nm)/Pd (x = 2.5 nm)/TAF (30 nm) architecture showed a 2.7-fold enhancement in the hydrogen-induced optical contrast at 1550 nm compared to Pd/TAF reference films, attributed to the hydrogen ion exchange between the Ti and Pd layers. The optimized structure, with a Pd thickness of x = 1.9 nm, exhibited hysteresis-free sensing behavior, a rapid response time ($t_{90}$ < 0.35 s at 4% $H_{2}$), and a detection limit below 10 ppm. It also demonstrated excellent selectivity with negligible cross-sensitivity to $CO_{2}$, $CH_{4}$, and CO, as well as high durability, showing less than 6% signal degradation over 135 hydrogenation cycles. These findings establish a scalable, room-temperature NIR hydrogen sensing platform with strong potential for deployment in automotive, environmental, and industrial applications.

## 1. Introduction

Hydrogen is a leading candidate for clean energy applications due to its high energy density, diverse production methods, and zero-emission combustion [1,2,3,4]. However, its flammability, wide explosive range, and lack of color or odor pose serious safety challenges, particularly during storage, transport, and end use [4,5]. These risks underscore the need for compact, fast, and highly sensitive hydrogen sensors that operate reliably under ambient conditions [3,6].

To meet these demands, various hydrogen detection strategies have been developed, including resistive, mechanical, optical, acoustic, electrochemical, and thermal sensing methods [4]. Among these, resistive sensors—especially based on palladium (Pd)—offer notable advantages such as their simple operation, low fabrication cost, ultra-sensitive LOD (down to a few ppb), and excellent stability [7,8,9,10]. However, their electrical nature poses spark risks in flammable environments. In contrast, optical hydrogen sensors offer intrinsic safety—such as being spark-free—by eliminating electrical components from the sensing area [11,12,13]. They also support non-contact and long-distance sensing, making them well-suited for a variety of applications in hazardous environments. These devices detect hydrogen through reversible changes in the optical properties—typically transmittance or reflectance [14,15,16]—of hydrogen-responsive metal films [4]. Palladium is also widely used in optical sensors due to its strong and reversible optical response at room temperature [7,17]. However, Pd-based systems often suffer from drawbacks such as hysteresis, slow kinetics across the α−β phase transition, and performance degradation over time due to cracking and delamination under cyclic stress [12,13,18,19].

To address these limitations, researchers have explored alloying Pd with other metals (e.g., Au, Ag, Co, Cu) and incorporating nanoscale features such as nanoholes, nanopatches, or nanowires [9,10,13,20,21,22]. While effective, these strategies often require complex fabrication processes that limit scalability and increase cost. Alternatively, integrating Pd with another hydrogen-active material in a bilayer configuration might offer a simpler and more scalable approach, and at the same time enhance performance by leveraging complementary material properties. For example, Boelsma et al. achieved hysteresis-free sensing using Pd–Hf bilayers, although their devices required elevated temperatures due to slow hydrogen diffusion at room temperature [23].

Titanium (Ti) is a promising candidate for such bilayer designs. It forms stable hydrides, exhibits excellent mechanical stability, and adheres well to Pd-reducing film delamination [19,24,25,26]. More importantly, Ti and Pd exhibit opposing dielectric responses to hydrogenation [24], which are particularly pronounced in the near-infrared (NIR) range. This contrast opens the possibility of an enhanced optical response through dielectric engineering. Despite this potential, Ti/Pd bilayers have remained largely unexplored for hydrogen sensing at telecom wavelengths near 1550 nm, a spectral region attractive for fiber-optic integration due to its low loss and wide commercial infrastructure [27,28,29].

In this work, we introduce a NIR optical hydrogen sensor based on a Ti/Pd bilayer capped with a gas-permeable polymer, Teflon AF (TAF), known to enhance hydrogen transport [30]. By optimizing the sensor (5 nm Ti/1.9 nm Pd/30 nm TAF), we achieved hysteresis-free sensing, sub-second response times $\left(t_{90}\left(s\right)<0.35\;s\right)$ at the lower flammability limit (4% $H_{2}$), detection limits below 10 ppm, and excellent long-term stability. The sensor also exhibited negligible cross-sensitivity to common interfering gases such as $CO_{2},CH_{4}$, and CO. These results establish the potential of Ti/Pd/TAF stacked films as a scalable, fiber-compatible, and high-performance platform for hydrogen sensing in safety and environmental monitoring system applications.

## 2. Materials and Methods

### 2.1. Materials

High-purity palladium (Pd, 99.95%) and titanium (Ti, 99.99%) were purchased from the Kurt. J Lesker Company (Jefferson Hills, PA, USA) and used to fabricate bilayer sensors. Teflon AF 2400 (TAF) obtained from Dupont (Wilmington, DE, USA) was used for polymer coating. Polymethyl methacrylate (PMMA), acetone (≥99.5%), and 2-propanol (≥99.9%) were purchased from Sigma-Aldrich (Saint Louis, MO, USA). Deionized (DI) water (18 MΩ cm) was used for all cleaning processes.

### 2.2. Thin-Film Deposition

Thin films were deposited in a custom-built electron-beam evaporation chamber (Pascal Technology, Fredericksburg, VA, USA) under a base pressure of $1\times 10^{-6}$ Torr. Glass substrates (1 cm × 1 cm) were cleaned using a sequential ultrasonic bath in acetone, 2-propanol, and DI water, each for 15 min, then dried under a nitrogen stream. A 5 nm Ti layer was deposited first, followed by a Pd layer with nominal thicknesses ranging from 1.9 to 2.5 nm. During deposition, the substrate was continuously rotated at 30 rpm to ensure uniform coverage. The deposition rate was maintained between 0.1 and 0.15 A/s and monitored via a quartz crystal microbalance (QCM, Brea, CA, USA).

### 2.3. Teflon AF and PMMA Deposition

After the metal deposition, a uniform ~30 nm-thick layer of Teflon AF 2400 (TAF) was applied using a Luxel vacuum-evaporation organic furnace at a pressure of <$1\times 10^{-6}$ mbar. For the PMMA coating, a 10 mg/mL PMMA–acetone solution was spin-coated onto the bilayer sensor at 5000 rpm for 120 s, forming a uniform ~100 nm-thick film [31]. The coating thickness was selected based on prior studies with the goal of providing complete coverage of the Pd surface without significantly hindering hydrogen diffusion [30,31,32]. TAF coating improves sensor performance by reducing the apparent activation energy for hydrogen absorption and desorption through interfacial chemical and electronic modification, while PMMA layers improve selectivity by limiting the permeation of interfering gases such as water and CO [30,31].

### 2.4. Structural and Morphological Characterization

The surface morphology was characterized by scanning electron microscopy (SEM) using ultra-high-resolution SEM (SU-9000; Hitachi, New Delhi, India) with a resolution of 0.4 nm at 30 kV. X-ray diffraction (XRD) measurements were performed using a powder diffractometer (XRD; Rigaku, Tokyo, Japan) with Cu-Kα radiation (λ=0.154 nm) to investigate the crystallographic structures of the films.

### 2.5. Optical Sensing and Measurement Setup

The hydrogen-sensing performance was evaluated using a custom-built optical setup [33]. The sensors were placed inside a custom fabricated vacuum chamber equipped with dual quartz optical windows for in situ optical measurements during the hydrogen exposure. The absolute pressure in the gas cell was monitored using three calibrated pressure transducers—two PX409-USBH transducers (Omega Engineering, Stamford, CT, USA) and a Baratron gauge (MKS Instruments, Andover, MA, USA)—enabling accurate readings from $\sim 2.7\times 10^{-6}$ up to 1.1 bar. By using diluted $4\%\;H_{2}\;or\;100\;ppm\;H_{2}$ balanced in $N_{2}$, we could control the partial $H_{2}$ pressure at as low as ~4 ppm or ~100 ppb. In the flow mode measurement, 4% $H_{2}$ in nitrogen mixture gases (Airgas, Radnor, PA, USA) was diluted with ultra-high-purity nitrogen gas from the Airgas company to targeted concentrations by a commercial gas blender (GB-103; MCQ Instruments, Roma, Italy). The gas flow rate was kept constant at 400 mL/min for all measurements. An unpolarized, collimated halogen light source (HL-2000, Ocean Optics, Orlando, FL, USA) was used to illuminate the sample at normal incidence. The transmitted light was collected into an optical fiber and directed to two spectrometers, USB4000-VIS-NIR-ES (Ocean Optics; 400–1000 nm) and DWARF-STAR (StellarNet, Tampa, FL, USA; 900–1650 nm), to record the transmission spectra across the visible and near-infrared regions. All the measurements were conducted at room temperature.

### 2.6. Finite-Difference Time-Domain Calculations

Finite-difference time-domain (FDTD) simulations were used to calculate the optical response of the Pd and Ti/Pd bilayer samples using commercial software (Ansys Academic Lumerical FDTD research, version 2024 R1.1). The geometric parameters of the Pd nano-particles were obtained from the SEM images and imported into the software. A mesh size of 2 × 2 × 2 nm was chosen. The refractive indices of the glass and TAF were chosen to be 1.5 and 1.27, respectively. The optical parameters of $Pd,PdH_{x},Ti,and\;TiH_{x}$ were obtained from previously reported experimental data [24].

## 3. Results and Discussion

### 3.1. Film Morphology and Structure

**Figure 1:**
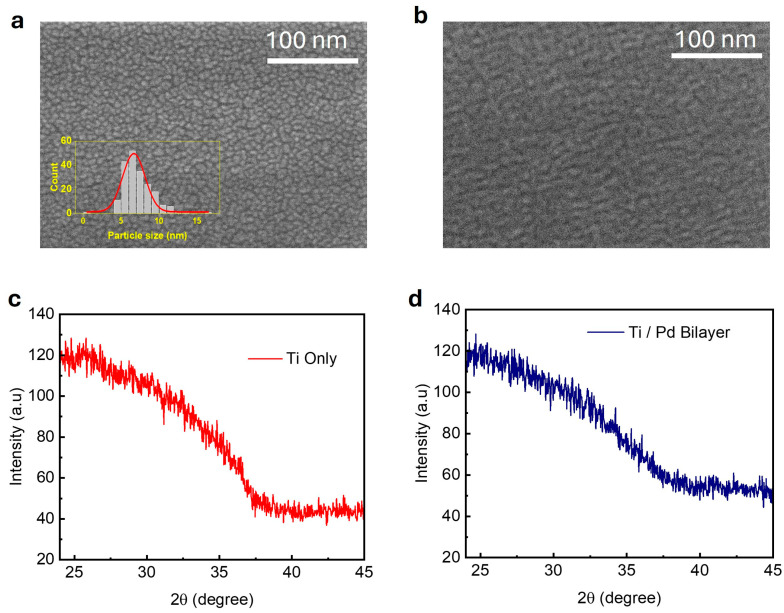
Scanning electron microscopy (SEM) images of (**a**) 2.5 nm Pd thin film, with estimated particle sizes shown in the inset, and (**b**) 5 nm Ti/2.5 nm Pd bilayer. X-ray diffraction (XRD) spectra of (**c**) 5 nm Ti film and (**d**) 5 nm Ti/2.5 nm Pd bilayer film.

The surface morphologies of the deposited films (Pd-only and Ti/Pd bilayer) were investigated using high-resolution SEM to understand the effect of the Ti underlayer on the Pd film growth. The 2.5 nm Pd-only film directly deposited on a glass substrate (Figure 1a) exhibited a discontinuous granular morphology, characterized by isolated sub-10 nm particles as shown in the Figure 1a inset. In contrast, when a similar thickness of Pd was deposited on a 5 nm Ti layer, the film exhibited significantly improved continuity, with evidence of particle coalescence (Figure 1b). This morphological transition suggests enhanced wetting and surface diffusion enabled by the Ti adhesion layer, which likely reduces nucleation barriers and promotes lateral film growth. These findings are strongly supported by the work of Verma et al. [25], who demonstrated that the surface topography of the Ti underlayer can control Pd grain morphology, leading to smoother, more continuous films with enhanced columnar structures and reduced void formation.

The X-ray diffraction (XRD) spectra (Figure 1c,d) of the 5 nm Ti-only and 5 nm Ti/2.5 nm Pd bilayer films show broad, featureless patterns, indicating a lack of long-range crystalline order within the detection limits. The absence of additional peaks in the bilayer suggests that the Pd overlayer did not significantly modify the structural phase of the Ti layer. Such behavior has been observed in prior studies of ultrathin metal films, where limited adatom mobility at room temperature during vapor-phase growth promotes island-like or discontinuous morphology, leading to nanoscale grains or amorphous structures that fall below the detection threshold of conventional XRD techniques [34,35].

### 3.2. Effect of Ti Underlayer in Pd/Ti Bilayer Films

#### 3.2.1. Enhanced NIR Optical Response

**Figure 2:**
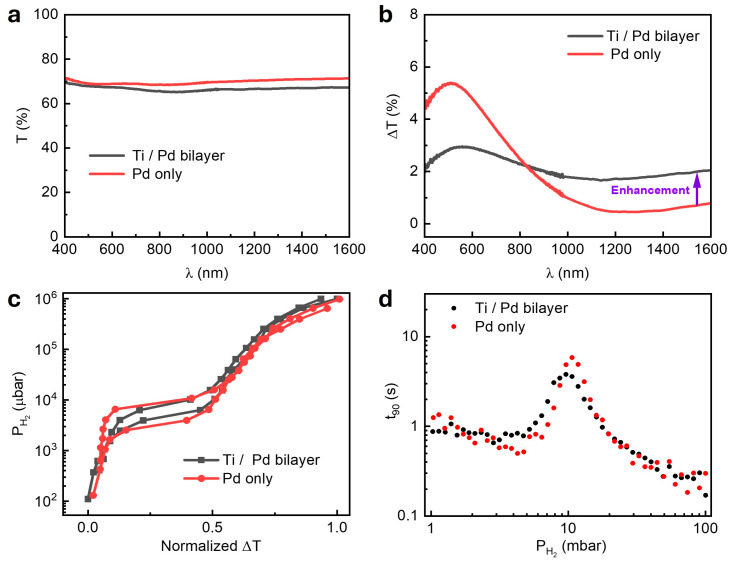
Optical transmission and sensing performances of 2.5 nm Pd/30 nm TAF thin films with and without a 5 nm Ti underlayer. (**a**) Experimental optical transmission spectra T(λ) in a vacuum (0 mbar $H_{2}$). (**b**) Hydrogen-induced change in transmission, ΔT(λ) = T_1000 mbar_ − T_0 mbar_, across the 400–1600 nm spectral range. (**c**) Normalized ΔT sorption isotherms at λ = 1550 nm. (**d**) Response time (t_90_) under hydrogen pressures ranging from 1–100 mbar.

The Ti/Pd bilayer exhibited a lower baseline transmission (T) than that of the Pd-only reference film across the 400–1600 nm wavelength range due to additional absorption by the Ti layer (Figure 2a). This trend was also captured by the FDTD simulations, which closely matched the experimental results (Figure S1). Upon hydrogen exposure, both films showed increased optical transmission, consistent with earlier Pd-based systems; however, the earlier studies were limited to wavelengths of shorter than 1000 nm [14,15,33]. The change in the transmission intensity at $P_{H_{2}}=1000\;and\;0\;mbar$, defined as $\Delta T\left(\lambda \right)=T_{1000}-T_{0},$ is attributed to hydrogen-induced changes in the electronic structures of the metals. Hydrogenation introduces extra electrons, raising the Fermi level and modifying the dielectric function. Palm et al. showed that this leads to the real and imaginary parts of the dielectric constant in Pd (Ti) decreases (increases), especially in the NIR region—resulting in an increase (decrease) in optical transmission [36,37,38,39].

The 2.5 nm Pd/30 nm TAF film exhibited a strong ΔT centered around 550–600 nm, but the response decreased rapidly with the increasing wavelength, dropping below 1% beyond 1000 nm (Figure 2b)—following a trend previously reported in Pd films [15]. In contrast, the 5 nm Ti/2.5 nm Pd/30 nm TAF structure maintained ΔT ~ 2% across the full spectral range. This behavior was further supported by the FDTD simulations and a theoretical transmission calculation for an absorbing film (hydrogenated Pd), described by the equation [14,15]

(1) $$T=\frac{16\;n_{s}\left(n^{2}+k^{2}\right)\;e^{-\frac{4\pi kd}{\lambda }}}{\left[{\left(n+n_{s}\right)}^{2}+k^{2}][{\left(1+n\right)}^{2}+k^{2}\right]}$$

where n and k are the effective refractive index and extinction coefficient of the Ti/Pd bilayer calculated from Figure S2a,d is the film thickness, λ is the wavelength, and $n_{s}$ is the refractive index of the substrate. Both the simulated and calculated spectra capture the overall spectral trend and key features of the experimental response, although some deviations can be observed in the magnitudes and positions of the peaks and dips (Figure S2b,c). Notably, at λ=1550 nm, the calculated ΔT of 2.07% closely matches the experimental value of 2.05%. This corresponds to a 2.7-fold enhancement over the reference film (ΔT = 0.76%) and underscores the advantage of Ti/Pd for near-infrared sensing. This enhancement is particularly significant for sensing near 1550 nm—a telecom-standard wavelength offering low-loss optical propagation and compatibility with photonic integrated circuits (PICs). Previous studies have demonstrated sub-ppm sensitivity for gases such as $NH_{3}\;and\;CH_{4}$ in this spectral region using PICs [29]. Our Ti/Pd/TAF sensor design extends this capability to hydrogen detection, offering a scalable and fiber-compatible optical sensing platform.

#### 3.2.2. Reduced Hysteresis in the Optical Sorption Isotherm

Hydrogen isotherms were obtained by monitoring the optical transmission at λ=1550 nm during the cyclic hydrogen exposure. While the data are presented at 1550 nm, the plateau pressures could have been extracted at any wavelength across the spectrum [13] (see also Figure S3a), their plateau pressures were essentially the same (Figure S3b), and their response times were identical (Figure S3c). The Ti/Pd/TAF structure exhibited a narrower hysteresis width ($P_{Hys}=3.4\;mbar$) and a smaller hysteresis index (HI=0.49) compared to the Pd/TAF reference film ($P_{Hys}=5.5\;mbar$ and HI = 0.911), as shown in Figure 2c and Table S1. Hysteresis width is defined as the pressure difference between absorption ($P_{Abs})$ and desorption ($P_{Des})$ $(P_{Hys}=P_{Abs}-P_{Des}$) [13], and the hysteresis index was calculated as $HI=\frac{P_{Abs}-P_{Des}}{{(P}_{Abs}+P_{Des})/2}$ [40]. This reduction in the hysteresis observed in the Ti/Pd/TAF structure is attributed to the role of the Ti underlayer through three synergistic effects. First, the Ti layer acted as a mechanical clamp—its rigidity constrained the palladium lattice during hydrogenation, thereby reducing the stress-induced deformations (e.g., cracking/delamination) that typically cause hysteresis in pure Pd films [19,41]. This mechanical stabilization resulted in smoother and more reversible hydrogen absorption, with a reduced optical transmission change (ΔT) below 800 nm (Figure 2b). The stronger ΔT response above 800 nm resulted from amplified dielectric contrast in the NIR region, where the Ti/Pd hydrides exhibited stronger and opposing optical responses [24]. Second, the Ti layer improved the overall film morphology. As shown in the SEM image (Figure 1b) and supported by SEM/XRD studies by Verma et al., the Ti underlayer promoted more continuous Pd growth with smaller grains [25]. This improved morphology enhanced the hydrogen diffusion uniformity, shortened the effective hydrogen diffusion path length, and minimized the localized trapping or strain accumulation, which contributes to reduced hysteresis behavior and potentially faster response times. Third, the interfacial interactions between the Ti and Pd likely led to the formation of nano-alloys or intermetallic phases at the interface. Studies on Ti-buffered Pd systems have shown that PdTi alloy formation is favored when the Pd layer is ultrathin [42]. These alloyed regions disrupt the long-range crystalline order in Pd, resulting in a more disordered or fine-grained microstructure. This structural transformation plays a critical role in hydrogen transport: smaller grains introduce a higher density of grain boundaries, which serve as efficient diffusion pathways due to their increased defect density and reduced atomic packing. These boundaries allow hydrogen to migrate more quickly through the film, minimizing delays between absorption and desorption and promoting more balanced kinetics. This interpretation is further supported by the smaller response-time ($t_{90}$) peak observed in the Ti/Pd bilayer compared to the Pd-only films (Figure 2d and Figure S4c). The response time (t_90_) was defined as the time needed for the optical signal to reach 90% of its total change.

#### 3.2.3. Faster Response and Enhanced Hydrogen Kinetics

Kinetic measurements were conducted by tracking the real-time changes in the optical transmission at the same wavelength during stepwise hydrogen pressure pulses from 100 to 1 mbar. The response time (t_90_) exhibited a peak near 10 mbar for both samples (Figure 2d) and corresponded to the α-β phase transition region in the palladium hydride. This behavior was previously reported in Pd-based optical and electrical hydrogen sensors [42,43,44,45]. This peak reflects slower kinetics due to phase coexistence and lattice rearrangement [20,46]. Notably, the 5 nm Ti/2.5 nm Pd/30 nm TAF structure reduced the peak response time to 3.8 s—about 35% faster than the 5.85 s observed for the 2.5 nm Pd/30 nm TAF reference film at 10 mbar. The effect was more pronounced for thinner Pd layers: at 2.25 nm, the peak, $t_{90}$, dropped from 5.19 s (Pd-only) to 2.52 s (Ti/Pd bilayer)—a 52% improvement (Figure S4c).

### 3.3. Optimization of Pd Thickness for High-Performance Sensing

**Figure 3:**
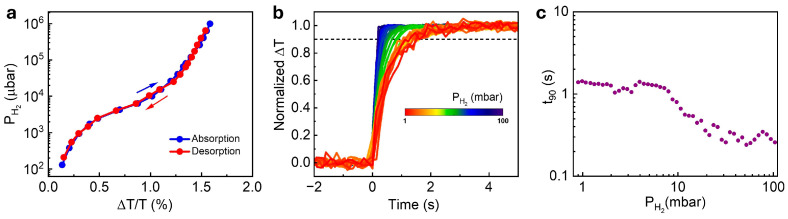
Optimized hydrogen sensing performance of 5 nm Ti/1.9 nm Pd/30 nm TAF film: (**a**) optical hydrogen sorption isotherms and (**b**) time-resolved normalized absorption responses of the sensor under hydrogen pressure from 100 to 1 mbar at room temperature. (**c**) Extracted response times (t_90_) of the sensors from panel (**b**) across the pressure range.

To optimize the performance of the Ti/Pd/TAF sensor, we reduced the Pd thickness to 2.25 nm and 1.9 nm while keeping the Ti underlayer and TAF capping thickness fixed. Among these configurations, the 5 nm Ti/1.9 nm Pd/30 nm TAF sensor demonstrated the best overall performance in terms of sensitivity, hysteresis suppression, and response time. Detailed results for this optimized structure are presented in Figure 3; data for the other configurations are provided in the Supplementary Materials (Figure S4).

The hydrogen-induced transmission change of the optimized sensor (5 nm Ti/1.9 nm Pd/30 nm TAF) was examined as previously described (Figure S5a). The corresponding isotherm, shown in Figure 3a, exhibits a minimal hysteresis width ($P_{Hys}<0.3\;mbar$) and hysteresis index (HI = 0.0345), indicating hysteresis-free behavior across the measured pressure range. From the time-resolved data, we observed no response-time peak—typically linked to the α–β phase transition in Pd—confirming that the transition was suppressed in this structure (Figure 3b,c). The sensor exhibited a rapid response ($t_{90}<0.35\;s$) at 4% $H_{2}$, the lower flammability limit, and maintained a <2 s response across the full 0.1–10% (1–100 mbar) $P_{H_{2}}$ range. This performance places the 5 nm Ti/1.9 nm Pd/30 nm TAF sensors among the faster thin-film optical hydrogen sensors operating at telecommunication wavelengths, comparable to more complex alloyed or nanostructured systems (see Table 1). Zeng et al. [42] similarly reported suppressed response time peaks in Ti/Pd electrical sensors with 2 nm Pd films, supporting the role of Pd thickness in mitigating the α–β phase transition; however, their devices exhibited significantly slower responses (>20 s) under comparable pressures.

**Figure 4:**
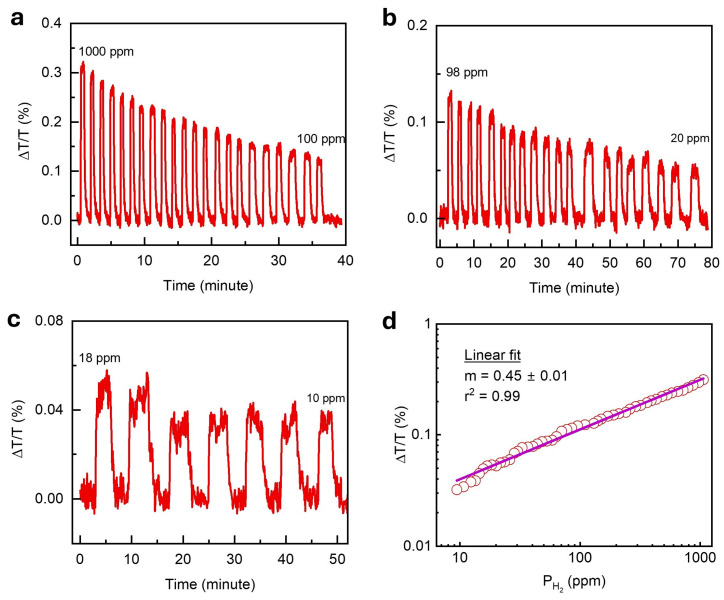
Limit of detection (LOD) measurements of the 5 nm Ti/1.9 nm Pd/30 nm TAF sensor. Transmission responses, ΔT/T, at λ = 1550 nm measured under decreasing hydrogen concentrations ranging from (**a**) 1000 ppm to 100 ppm, (**b**) 98 ppm to 20 ppm, and (**c**) 18 ppm to 10 ppm. (**d**) The measured optical sensitivity (ΔT/T) as a function of low hydrogen concentration, derived from panels (**a**–**c**), follows Sievert’s power law fit (solid line).

The limit of detection (LOD) is a fundamental metric that represents the lowest hydrogen concentration a sensor can reliably detect. To determine the LOD of the optimized 5 nm Ti/1.9 nm Pd/30 nm TAF sensor, we extended the measurements to below 1 mbar—beyond the range shown in Figure 3—by gradually reducing the hydrogen concentration from 1000 ppm (0.1%) down to 10 ppm and recording the corresponding optical transmittance response (Figure 4a–c). For the concentrations below 100 ppm, the sensor was tested using $4\%\;H_{2}$ diluted in $N_{2}$. Even at 10 ppm, the signal remained clearly detectable, with reversible on–off cycling confirming reliable performance at ultra-low concentrations. The pressure-dependent sensor’s response (ΔT/T) followed Sievert’s law behavior, $\frac{\Delta T}{T}\propto {\left(P_{H_{2}}\right)}^{m}$ [47], with the exponent, m, extracted from fitting the data. A slope of m = 0.448 ± 0.007 was obtained for $P_{H_{2}}\leq 1000\;ppm$ (Figure 4d), while a slightly lower value of m = 0.37 ± 0.009 was across the full measured $P_{H_{2}}$ range (Figure S5b). These results indicate that hydrogen uptake in a Ti/Pd bilayer is governed by solubility-driven absorption dynamics, consistent with metal–hydride thermodynamics.

**Figure 5:**
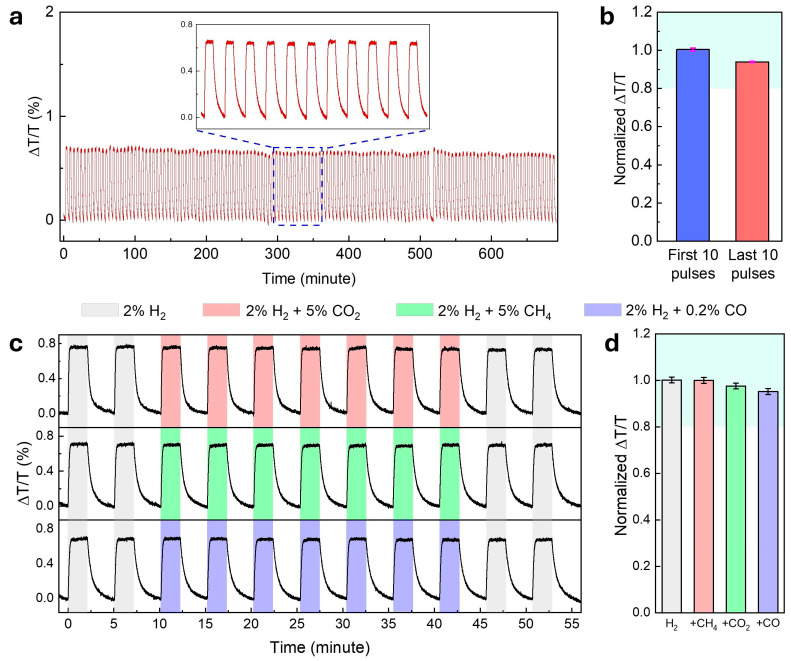
Long-term stability and effects of interfering gases on the 5 nm Ti/1.9 nm Pd/30 TAF sensor. (**a**) Optical response (ΔT/T) over 135 hydrogen cycles (2/3 min of loading/unloading) with 2% $H_{2}$ balanced in $N_{2}$ gas with a total flow of 400 mL/min. (**b**) Comparison of normalized response (ΔT/T) amplitude for the first and last 10 pulses in figure (**a**). (**c**) Time-resolved optical responses (ΔT/T) of the sensor to 2 pulses of 2% $H_{2}$ followed by 7 pulses of $2\%\;H_{2}+5\%\;CO_{2},$ $2\%\;H_{2}+5\%\;CH_{4},2\%\;H_{2}+0.2\%\;CO$ and a final 2 pulses of 2% $H_{2}\;$ in $N_{2}$ as a gas carrier. (**d**) Normalized sensor signal intensity compared with the reference signal obtained in the 2% $H_{2}\;in\;N_{2}$ gas flow extracted from (**c**). The error bars denote the standard deviation from 7 cycles.

The long-term stability and durability of the optimized Ti/Pd/TAF sensor were evaluated through over 135 consecutive hydrogenation and dehydrogenation cycles using 2% $H_{2}\;in\;N_{2}$ under flow-mode measurements. As shown in Figure 5a, the optical signal (ΔT/T) at λ=1550 nm remained stable over the entire 700 min test duration, exhibiting no baseline drift or signal loss that was observed, indicating excellent long-term stability. This exceptional cycling stability is attributed to the Ti underlayer, which reinforced the mechanical integrity by mitigating the volume expansion in the Pd layer, thereby preventing delamination and preserving the sensing performance over extended use [19]. Furthermore, a direct comparison of the response amplitude between the first and last 10 cycles revealed a gradual sensitivity decrease of less than 6%, with minimal error bars (Figure 5b). This minor degradation is attributed to the buildup of residual strain from repeated lattice expansion and contraction. Such strain alters the energy landscape for hydrogen absorption, increasing the activation barrier in later cycles [48]. However, evaluating the long-term performance of the sensor would require cyclic testing under accelerated conditions and exposure to various gas environments, including all potential interfering gases—an effort that falls outside the scope of this report [49].

Sensor selectivity—referring to the ability to detect a target analyte in the presence of other gases—and resistance to chemical poisoning are critical challenges in gas sensor development. An ideal hydrogen sensor should respond exclusively to hydrogen without interference from other gases commonly present in the environment. To evaluate the selectivity of the optimized Ti/Pd/TAF sensor, we exposed it to 2% $H_{2}\;in\;N_{2}$, both alone and in a mixture with 5% $CO_{2}$, 5% $CH_{4}$, or 0.2% CO. As shown in Figure 5c and in the normalized response in Figure 5d, the sensor exhibited consistent ΔT/T signals across all gas compositions, with no observable degradation during the exposure to the mixed atmospheres. Although TAF is known for its high gas permeability and poor selectivity [50], the observed selectivity toward $CO_{2}$ and $CH_{4}$ may be attributed to the intrinsic resistance of Pd [13]. However, it cannot fully protect against CO [13,31]. To overcome this drawback, a PMMA layer of 100 nm thickness was coated on top of the Ti/Pd/TAF, as PMMA has been demonstrated to be an excellent hydrogen-selective membrane [30,51]. Notably, the addition of the PMMA coating did not affect the response time compared to the uncoated sensor [31].

While this study did not directly evaluate sensor performance under variable temperature and humidity conditions, our previous reports [13,31] have demonstrated that PMMA coating effectively mitigates interference from water vapor. Additionally, our results (Figure 5c,d) show that PMMA provides protection against common toxic gases such as CO. However, PMMA alone does not fully suppress degradation caused by oxygen exposure [9,48]. To further enhance environmental stability, a tandem protective layer can be employed—specifically, a PMMA base coating followed by a thin polyvinyl alcohol (PVOH) film. This bilayer strategy significantly reduces oxygen interference, as PVOH acts as an excellent oxygen barrier [52]. About the temperature effect, elevated temperatures accelerate hydrogen uptake kinetics in Pd-based sensors—resulting in faster response and recovery—but they also reduce sensitivity [13,48]. This reduction occurs because hydrogen absorption in palladium is an exothermic process, so higher temperatures shift the equilibrium toward $H_{2}$ release. As a result, less hydrogen is absorbed at a given concentration, diminishing the sensor’s signal. In order to mitigate the temperature effect, the temperature of the sensor is optimized and uniformly fixed at a designed temperature [53].

**Table 1:** To contextualize the performance of our Ti/Pd/TAF-based optical hydrogen sensor, we compared its key metrics—response time, limit of detection (LOD), and hysteresis behavior—with other state-of-the-art optical hydrogen sensors operating at room temperature. n.a. = not addressed.

Sensing Platform	$t_{90}\left(s\right)$ (@ 40 mbar)	$t_{90}\left(s\right)$ (@ 1 mbar)	LOD (ppm)	Hysteresis-Free?	Ref.
Ti/Pd/TAF film	≤0.35	<2	<10 ppm	Yes	This work
$Pd_{80}Co_{20}NP$	≤0.15	0.85	2.5	Yes	[13]
PdAu nano-particles @ PTFE/PMMA (100 × 25 $\left(nm^{2}\right)$)	≤0.3	1	<1000 (estimated)	Yes	[30]
Pd nano-disk array	>10	-	50	n.a.	[45]
Pd bilayer lattices	~900	55	-	n.a.	[44]
PdAuCu nano-particles	0.4	-	5	Yes	[21]
PdAu nanostructures	40	-	-	yes	[54]
Pd strip	-	20	10	n.a.	[55]
PdY film	6	-	1000	n.a.	[56]
Pd/SiO2/Au	3	-	5000	n.a.	[57]
Pd/Au film	4.5	-	-	n.a.	[58]

## 4. Conclusions

We have demonstrated a simple and effective approach to develop a hysteresis-free, fast-response, highly sensitive, and long-term-stable hydrogen sensor without relying on complex alloying or nanostructuring. By exploiting the opposing dielectric responses of Ti and Pd to hydrogenation, the sensor achieved strong optical contrast at the telecom wavelength of 1550 nm. The optimized structure responded within 0.35 s at 4% hydrogen, detected concentrations of below 10 ppm, and maintained consistent performance over 135 hydrogenation cycles with less than 6% signal degradation. The sensor also exhibited negligible cross-sensitivity to common interfering gases such as $CO_{2},CH_{4}$, and CO. These findings highlight a promising path toward fiber-compatible, spark-free optical sensors for real-time hydrogen monitoring in safety-critical and industrial environments.

## Data Availability

The data supporting the findings of this study are available from the corresponding author upon reasonable request.

## Supplementary Materials

The following supporting information can be downloaded at: https://www.mdpi.com/article/10.3390/nano15141105/s1.

## Abbreviations

The following abbreviations are used in this manuscript: TAF, Teflon AF 2400; PMMA, Polymethylmethacrylate; SEM, scanning electron microscopy; XRD, X-ray diffraction; LOD; limit of detection.
