# Supplementary material for: High-Speed and Hysteresis-Free Near-Infrared Optical Hydrogen Sensor Based on Ti/Pd Bilayer Thin Films
Source: Nanomaterials (Basel). 2025 Jul 16;15(14):1105. doi: 10.3390/nano15141105 (PMC12299049; doi:10.3390/nano15141105)
Supplement: Supplementary file 1 [file nanomaterials-15-01105-s001.zip › nanomaterials-3753103-supplementary.pdf]

*nanomaterials*

MDPI

*Supplementary Materials*

# High-Speed and Hysteresis-Free Near-Infrared Optical Hydrogen Sensor Based on Ti/Pd Bilayer Thin Films

**Ashwin Thapa Magar [1,*], Tu Anh Ngo [1], Hoang Mai Luong [2], Thi Thu Trinh Phan [3], Minh Tuan Trinh [3], Yiping Zhao [1] and Tho Duc Nguyen [1,*]**

[1] Department of Physics and Astronomy, University of Georgia, Athens, GA 30602, USA; anhngo@uga.edu (T.A.N.); zhaoy@uga.edu (Y.Z.)

[2] Department of Electrical Engineering, Faculty of Engineering, Chulalongkorn University, Bangkok 10330, Thailand; hoang.l@chula.ac.th

[3] Department of Chemistry and Biochemistry, Utah State University, Logan, UT 84322, USA; phan.trinh@usu.edu (T.T.T.P.); t.trinh@usu.edu (M.T.T.)

* Correspondence: ashwin.thapa@uga.edu (A.T.M.); ngtho@uga.edu (T.D.N.)

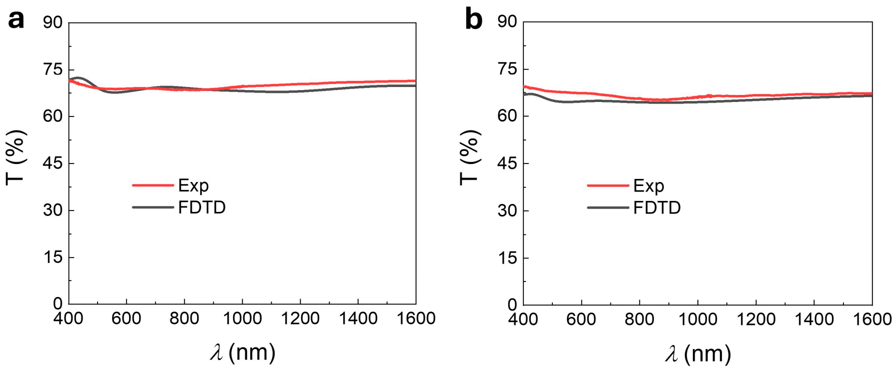


Figure S1. Comparison between experimental (Exp) and finite-difference time-domain (FDTD) simulated optical transmission spectra for (a) 2.5 nm Pd/TAF, and (b) 5 nm Ti/2.5 nm Pd/TAF thin film configurations.

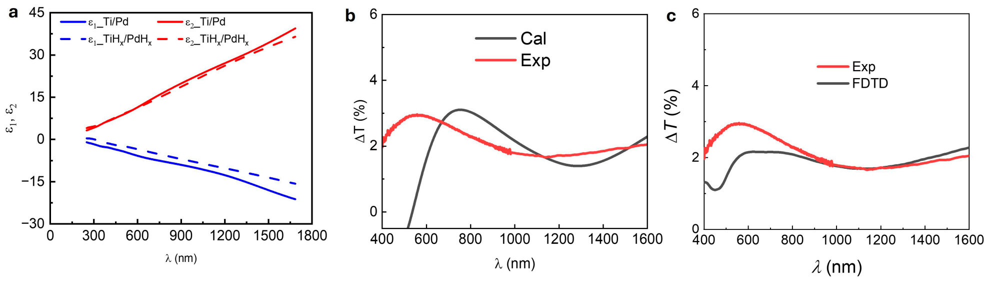


Figure S2. (a) Dielectric functions of the metal and its hydride for the 5 nm Ti/2.5 nm Pd bilayer structure, calculated using S1 equation. (b) Hydrogen-induced optical transmission change (ΔT) obtained from experimental measurements (Exp) and theoretical calculations (Cal) based on the equation 1 described in the main text. (c) Comparison between simulated (FDTD) and experimental ΔT spectra for the same bilayer sample.

 https://doi.org/10.3390/nano15141105

Dielectric functions of the metals Pd and Ti along with their respective hydrides $PdH_x$ and $TiH_x$ were extracted from literature data [1] . To estimate the optical behavior of the bilayer (5 nm Ti/2.5 nm Pd) structure, the effective dielectric function was calculated using a weighted average approach (Eq. s1), appropriate for thin films whose total thickness is much smaller than the wavelength of interest [2]. The expression for the effective dielectric function is given by:

$$\varepsilon_{eff} = \frac{d_{Pd} \times \varepsilon_{Pd} + d_{Ti} \times \varepsilon_{Ti}}{d_{Ti} + d_{Pd}} \quad \text{(S1)}$$

Where $d_{Pd}$ and $d_{Ti}$ represent the thickness of the Pd and Ti layer, $\varepsilon_{Pd}$ and $\varepsilon_{Ti}$ denote their respective dielectric constants. The weighted average model (Equation S1) assumes distinct, homogeneous Ti and Pd layers with abrupt interfaces and no intermixing. However, in reality, interfacial diffusion and surface roughness may cause deviations from this ideal structure [3]. Additionally, hydrogenation may further enhance intermixing or lead to the formation of intermetallic phases [4], altering the bilayer's optical properties. Collectively, these factors likely contribute to the discrepancies observed between the FDTD-simulated and experimental spectra below 900 nm, particularly in the magnitude of spectral peaks and dips (Figure S2c). Nonetheless, the strong agreement in spectral trends above 900 nm and the overall correspondence support the model's utility as a first-order approximation for predicting the NIR optical response of the Ti/Pd bilayer system.

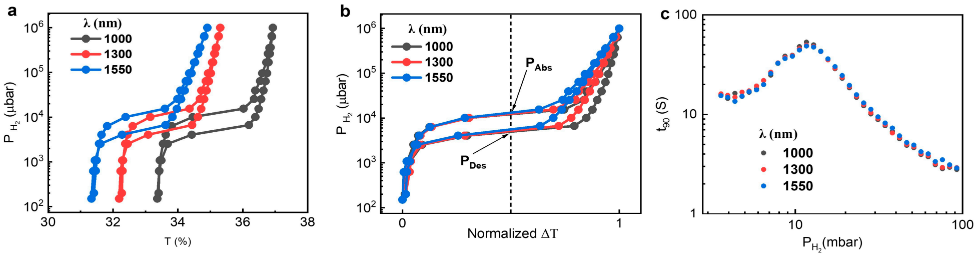


Figure S3. (a) Optical hydrogen sorption isotherms of the 5 nm Pd thin film measured at various near-infrared (NIR) wavelengths. (b) Normalized isotherms corresponding to the wavelengths shown in panel (a). (c) Hydrogen response times at the corresponding NIR wavelengths.

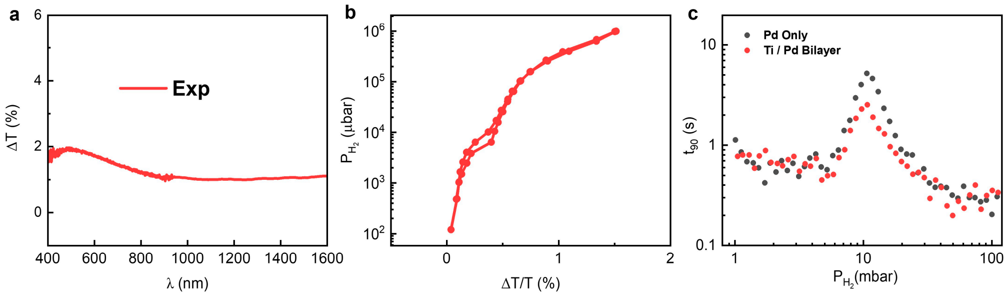


Figure S4. Sensing performance of the 5 nm Ti/2.25 nm Pd/30 nm TAF sensor (a) Hydrogen-induced optical transmission changes ($\Delta T(\lambda) = T_{1000\ mbar} - T_{0\ mbar}$) across the spectral range of 400 – 1600 nm. (b) Optical hydrogen sorption isotherm at $\lambda$ = 1550 nm. (c) Comparison of response times between the Ti/Pd/TAF sensor and Pd/TAF reference film under hydrogen pressure ranging from 1 to 100 mbar.

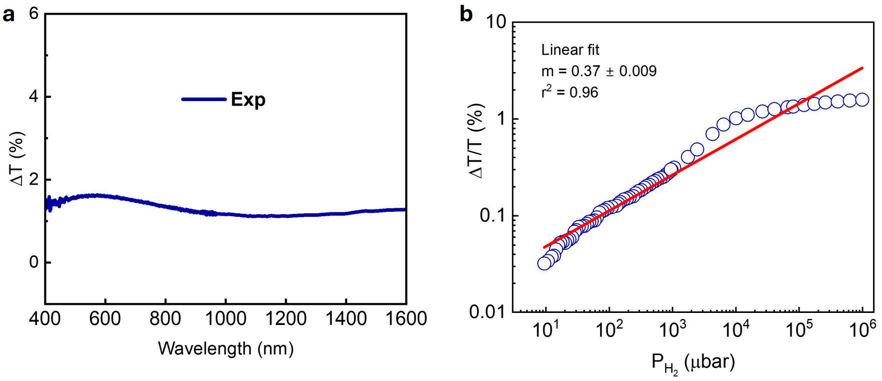


Figure S5. Optimized 5 nm Ti/1.9 nm Pd/30 nm TAF sensor (a) Hydrogen-induced optical transmission changes ($\Delta T(\lambda) = T_{1000\ mbar} - T_{0\ mbar}$) of the across the 400 – 1600 nm spectral range (b) Limit of detection (LOD) of the optimize sensor, where the sensor response follows sievert's power law, expressed as, $\frac{\Delta T}{T} \propto (P_{H_2})^m$ [5].

Table S1. Hysteresis Metrics for Ti/Pd/TAF and Pd/TAF Hydrogen Sensor Structures

| Devices structure | Hysteresis Width ( $P_{Hys} = P_{Abs} - P_{Des}$) [6] | Hysteresis Index $HI = \frac{P_{Abs} - P_{Des}}{(P_{Abs}+P_{Des})/2}$ [7] |
|---|---|---|
| 2.5 nm Pd / TAF | 5.5 mbar | 0.911 |
| 5 nm Ti / 2.5 nm Pd / TAF | 3.4 mbar | 0.49 |
| 5 nm Ti / 2.25 nm Pd / TAF | 2.88 mbar | 0.38 |
| 5 nm Ti / 1.9 nm Pd / TAF | < 0. 3 mbar | 0.0345 |

## References


1. Palm, K.J., et al., *Dynamic Optical Properties of Metal Hydrides.* ACS Photonics, 2018. **5**(11): p. 4677-4686.
2. Hu, F.G., J. Song, and T. Kamgaing. *Modeling of multilayered media using effective medium theory*. in *19th Topical Meeting on Electrical Performance of Electronic Packaging and Systems*. 2010.
3. Verma, N., et al., *Controlling morphology and texture of sputter-deposited Pd films by tuning the surface topography of the (Ti) adhesive layer.* Surface and Coatings Technology, 2019. **359**: p. 24-34.
4. Zeng, X.Q., et al., *Hydrogen responses of ultrathin Pd films and nanowire networks with a Ti buffer layer.* Journal of Materials Science, 2012. **47**(18): p. 6647-6651.
5. Caravella, A., et al., *Sieverts Law Empirical Exponent for Pd-Based Membranes: Critical Analysis in Pure H2 Permeation.* The Journal of Physical Chemistry B, 2010. **114**(18): p. 6033-6047.
6. Luong, H.M., et al., *Sub-second and ppm-level optical sensing of hydrogen using templated control of nano-hydride geometry and composition.* Nature Communications, 2021. **12**(1): p. 2414.
7. Rong, Y., et al., *Tunable hysteresis effect for perovskite solar cells.* Energy & Environmental Science, 2017. **10**(11): p. 2383-2391.
